# The Clinical Characteristics and Treatment Outcomes of Mesonephric Tumours of the Uterine Cervix: A Systematic Review and Proposal of Embryologically-Oriented Surgical Resection

**DOI:** 10.3390/jcm14010117

**Published:** 2024-12-28

**Authors:** Mohammad Daas, Christina Pappa, Dana Shibli, Abdallah Al-Ani, Sunanda Dhar, Sanjiv Manek, Ahmad Sayasneh, Moiad Alazzam

**Affiliations:** 1 Basildon and Thurrock University Hospital, Basildon SS16 5NL, UK; mo.daas1996@gmail.com; 2 Oxford University Hospitals, NHS Foundation Trust, Oxford OX3 9DU, UK; sunanda.dhar@ouh.nhs.uk (S.D.); sanjiv.manek@ouh.nhs.uk (S.M.); research@gynaefellow.com (M.A.); 3 Jordan University Hospital, Amman 11942, Jordan; shiblidana76@gmail.com; 4 King Hussein Cancer Center, Amman 11941, Jordan; abdallahalany@gmail.com; 5 Guy’s and St Thomas’ NHS Foundation Trust, London SE1 7EH, UK; ahmad.sayasneh@gstt.nhs.uk

**Keywords:** mesonephric neoplasm, mesonephric carcinoma, uterine cervical neoplasms

## Abstract

**Background/Objectives**: Cervical mesonephric adenocarcinomas (MNACs) are among the rarest neoplasms of the female genital tract. Unlike the majority of cervical cancers, which are predominantly squamous in origin and strongly associated with HPV seropositivity, MNACs are distinct in both histology and pathophysiology. Despite their unique characteristics, MNACs have historically been managed in parallel with squamous cell carcinomas, resulting in a lack of optimised, evidence-based treatment protocols. In this systematic review, we aim to evaluate the current management strategies for MNACs and their associated clinical outcomes. Additionally, we critically appraise existing surgical and adjuvant therapies and propose embryologically oriented surgical techniques to achieve optimal tumour resection. **Methods**: We performed a systematic search across the MEDLINE, CENTRAL, EMBASE, and ClinicalTrials.gov databases from 1960 to June 2024. The search strategy employed a combination of keywords and MeSH terms, including “Uterine Cervical Neoplasms” [MeSH], “mesonephric tumour”, “mesonephric neoplasm”, and “mesonephric cancer”. All relevant publications, including case reports and case series, were considered. **Results**: A total of 49 publications were finally included in the analysis, involving a thorough description of 91 MNAC cases. Most patients had stage I disease (70.8%) (*n* = 51). Hysterectomy was performed in 77 patients. The median follow-up was 29 months (range 1–199 months). Disease recurrence was observed in 35.2% (*n* = 25) of the cases, with the median disease-free survival (DFS) being 24 months (range 1–199). At the follow-up, 64.8% (*n* = 46) of patients remained in remission irrespective of the treatment modality, while 27.4% (*n* = 20) died due to disease progression. **Conclusions**: Mesonephric neoplasms of the uterine cervix are rare and clinically aggressive cancers that signify poor prognosis. Accurate identification and effective management can be challenging due to their particular anatomic and immunohistochemical characteristics. Therefore, a more tailored embryological-based approach should be considered for an optimal oncologic outcome.

## 1. Introduction

Mesonephric adenocarcinoma (MNAC) is a rare neoplasm accounting for less than 1% of cervical carcinomas [1]. It derives from the remnants of the mesonephric (Wolffian) duct, a precursor to the male genital tract, which typically regresses in females in the absence of androgen stimulation. However, in approximately 22% of females, remnants of the duct persist, potentially giving rise to mesonephric cysts, hyperplasia, or carcinoma [2,3]. The accurate assessment of MNAC incidence has been hampered by historical diagnostic inaccuracies, inadequate reporting (often mistaken for mesonephric hyperplasia), misclassification as clear cell carcinoma or yolk sac tumour, and the overall scarcity of reported cases [4]. The existence of a wide range of differential diagnoses further complicates the accurate diagnosis of MNAC [5].

Histologically, MNACs show significant variability in phenotypic architectural patterns, with cell morphology often differing within a single tumour, leading to potential misinterpretation of tumour specimens. Characteristic morphological features of MNAC include tubular, ductal, retiform, solid, spindle cell, and sex-cord-like patterns, as well as combinations of these architectural features [6]. Given this histological variability, accurate diagnosis often necessitates the examination of immunohistochemical profiling [3].

Due to the limited number of cases and the paucity of evidence, there is little consensus on the optimal management and prognosis of MNAC [7]. While some reports describe MNAC as following an indolent course with frequent recurrences, others suggest a more aggressive clinical trajectory associated with poor survival outcomes [8,9,10,11,12,13]. The majority of MNAC cases are treated surgically, with either simple or radical hysterectomy, bilateral salpingo-oophorectomy, and pelvic lymphadenectomy being common approaches [12]. The preference for surgical management is based on the observation that the surgical resection of MNACs is associated with better survival outcomes, particularly due to the high rates of metastasis to pelvic and para-aortic lymph nodes [14]. Additionally, many cases include the use of adjuvant chemoradiotherapy, either to mirror the treatment of similar carcinomas of the female genital tract or to mitigate the risk of tumour spillage during surgery [12,14,15,16]. The current surgical technique for radical hysterectomy restricts vaginal resection to approximately 10–20 mm below the caudal margin of the cervix according to the extent and radicality of resection [17,18,19]. This limited resection poses a challenge, particularly in cases of MNAC neoplasm infiltration, where more extensive resection of the vaginal cuff may be necessary. The inability to achieve a more radical resection increases the risk of positive vaginal margins, often necessitating the use of adjuvant therapies such as chemotherapy or radiotherapy to reduce the risk of recurrence. Therefore, incorporating elements of a more extensive vaginal resection may be worth considering in selected cases of MNAC, where the pattern of spread and embryological origins suggests a higher likelihood of upper vaginal involvement.

Given the current lack of optimised treatment modalities, there is a clear need for surgical approaches tailored to the embryological origin and invasion patterns of MNAC within the female genital tract. In this systematic review, we conducted a critical evaluation of all reported cases of MNAC to assess survival outcomes across different treatment strategies and to analyse the prognosis in cases with positive vaginal margins.

## 2. Materials and Methods

### 2.1. Literature Search Strategy

The literature search, primary and secondary screenings, and full-text analysis were conducted in accordance with the Preferred Reporting Items for Systematic Reviews and Meta-Analyses (PRISMA) guidelines [17]. A comprehensive search was performed using the MEDLINE (PubMed), CENTRAL, and ClinicalTrials.gov databases to identify all studies on mesonephric neoplasms in the female genital tract up to 30 June 2024. The search utilised a combination of keywords and MeSH terms: (“mesonephric tumour” OR “mesonephric tumour” OR “mesonephric cancer” OR “mesonephric neoplasm” OR “mesonephric adenocarcinoma”) AND (“Cervix” OR “Uterine Cervix” OR “Female genital tract” OR “female reproductive system”). Two independent authors (MD and AA) conducted the search and removed duplicate publications, retaining only the relevant studies. Articles were screened in two stages: initially by title and subsequently by abstract. Discrepancies between the authors were resolved through discussion and consultation with the senior authors (CP and MA). Full-text analysis was performed on the selected articles to extract relevant data for the final review.

### 2.2. Inclusion and Exclusion Criteria

We included studies reporting on mesonephric neoplasms of the uterine cervix that provided adequate descriptions of patient characteristics, treatment modalities, follow-ups, and outcomes. Eligibility criteria were structured according to the PICOS format (Population, Intervention, Comparison, Outcomes, and Study design). Eligible studies included patients diagnosed with cervical mesonephric neoplasms (P), who were treated either surgically (including simple hysterectomy or radical hysterectomy with or without bilateral salpingo-oophorectomy, pelvic and/or para-aortic lymphadenectomy, or local excision) or with chemotherapy, radiotherapy, or both as primary or adjuvant treatment (I). The presence of a comparator was optional considering the rarity of the condition, and we therefore included all treatments mentioned in the intervention (C). Outcomes of interest (O) included follow-up periods, recurrence rates, and survival status such as no evidence of disease (NED), dead with disease (DOD), or alive with disease (AWD). Given the rarity of MNAC cases, case reports and case studies were also included (S). Exclusion criteria encompassed non-English publications, reviews, letters to editors, editorials, animal studies, study protocols, abstracts, and brief correspondences.

### 2.3. Data Extraction

Two independent authors (MD and AA) extracted data from each study. The extracted information included first author, year of publication, stage at diagnosis, primary and secondary treatments, status of vaginal margins (extension/progression), use and type of adjuvant treatment, follow-up periods, presence and location of recurrence, overall survival outcomes, and time intervals from initial treatment to recurrence or death.

### 2.4. Risk of Bias Assessment

A risk of bias assessment for the included studies was conducted by two independent authors (MD and CP) using the Joanna Briggs Institute (JBI) Critical Appraisal Checklists for Case Reports and Case Series to ensure methodological quality and reliability of findings. The checklists include questions that assess specific domains of case reports and case series to assess the potential risk of bias. Any disagreements between reviewers were discussed with a third author (MA) and resolved by consensus.

### 2.5. Statistical Analysis

Statistical analyses were performed using SPSS version 30. Descriptive statistics were used to analyse the demographic and clinical characteristics of patients. Continuous variables are presented as mean ± standard deviation or median (range), and categorical variables are presented using frequency (*n*) and percentage (%).

Our analysis exclusively included case reports and case series; therefore, we acknowledge that the heterogeneity of the reported data can harbour significant variability and bias in the reported outcomes. Considering the rarity of the cases, it is unlikely that a future study might be able to include enough cases to conclude significant differences in treatment modalities and outcomes. Hence, we decided to perform a further survival analysis and Cox regression analysis to estimate the influence of the different variables on survival as a conservative estimate. The Kaplan–Meier method was used to perform disease-free survival (DFS), and overall survival (OS) analyses, and log-rank tests were used to compare survival rates. Potential risk factors for recurrence and mortality were analysed and assessed using univariate and multivariate Cox regression analysis. A *p*-value < 0.05 was considered statistically significant.

We would like to emphasise that the results should be interpreted with caution due to the high risk of bias deriving from the nature of the included studies.

## 3. Results

### 3.1. Search Results

**Figure 1:**
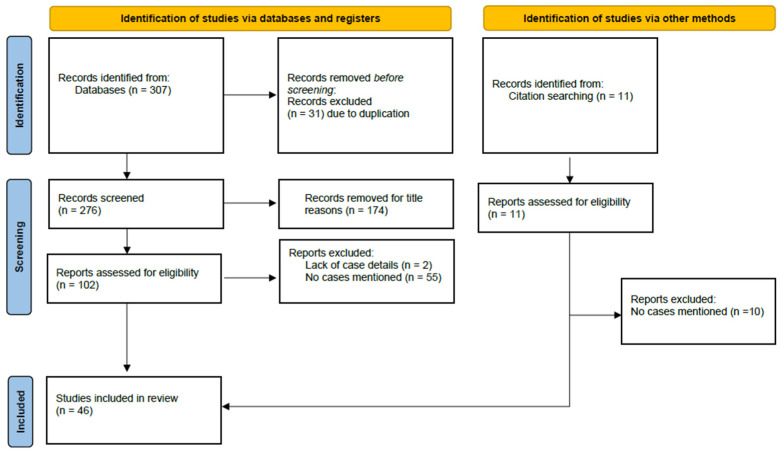
PRISMA flow diagram of screening and selection process.

A total of 318 relevant articles were initially identified. Following the removal of 31 duplicate entries, primary and secondary screenings excluded 174 additional articles. The remaining 113 articles underwent a full-text analysis to assess their eligibility. Ultimately, 48 studies were included in the qualitative synthesis. A summary of the selection process is shown in Figure 1.

### 3.2. Characteristics of Eligible Studies

The review included 48 studies between 1960 and 2024. Of these studies, 7 were case series and 41 were case reports. Overall, 91 patients with confirmed cervical mesonephric tumours were reported in the studies. The baseline clinical characteristics, treatment modalities, and outcomes are documented in Table 1. Based on the geographical region, 15 studies were reported from North America (46 cases), 15 from Europe (22 cases), 17 from Asia (22 cases), and 1 from South America (1 case).

### 3.3. Risk of Bias Assessment

We assessed 34 studies to have a low risk of bias, 8 with a moderate risk of bias, and 6 with a high risk of bias based on the JBI critical appraisal checklists for case series and case reports. The risk of bias quality assessment of the studies is presented in Table A1.

### 3.4. Patient Characteristics

A total of 91 cases were analysed. The stage at diagnosis was available for 72 cases, of which 70.8% (*n* = 51) were stage I, 15.4% (*n* = 11) were stage II, 6.9% (*n* = 5) were stage III, and 6.9% (*n* = 5) were stage IV. The most commonly recorded sub-stage among the studied population was stage IB, occurring in 40 patients (55.6%). Patient characteristics, treatment details, and follow-up outcomes are reported in Table 2.

In terms of adopted surgical management modalities, 61.5% (*n* = 56) of all included cases underwent total hysterectomy whether open, laparoscopic, robotic, or vaginal, while 23.1% (*n* = 21) underwent radical abdominal hysterectomy (RAH). In addition, 76.9% (*n* = 70) had bilateral or unilateral salpingo-oophorectomy (USO or BSO), and 50.5% (46) had pelvic lymph node dissection (PLND). Vaginal margins were reported in 21 cases, of which only 10 cases demonstrated positive invasion to the vaginal walls.

Among those with documented adjuvant therapy (*n* = 45), 15.6% (*n* = 7) were subjected to adjuvant chemotherapy (aCT), 53.3% (*n* = 24) had adjuvant radiotherapy (aRT), while 31.1% (*n* = 14) had both. Dual management, including surgery and adjuvant chemoradiotherapy, was observed in (*n* = 35) 35.7% of the patients with available data for both treatment modalities. Radiotherapy alone was given to eight patients.

Follow-up data were available in 71 cases. The median follow-up was 29 months (range 1–199 months). Disease recurrence was observed in 35.2% (*n* = 25) of the cases, with the median disease-free survival (DFS) being 24 months (range 1–199). The recurrence rate was noted to be 19.7% (*n* = 13) in stage I, 75.8% (*n* = 5) in stage II, 3% (*n* = 2) in stage II, and 3% (*n* = 2) in stage IV of disease.

The pelvis was the most common location for local tumour recurrence (*n* = 6), and lung metastasis was the most common site for distant metastasis (*n* = 7). The recurrence rate for patients subjected to CT was 42.1%, while patients receiving RT or bi-modal treatment had recurrence rates of 30% and 28.1%, respectively.

**Figure A1:**
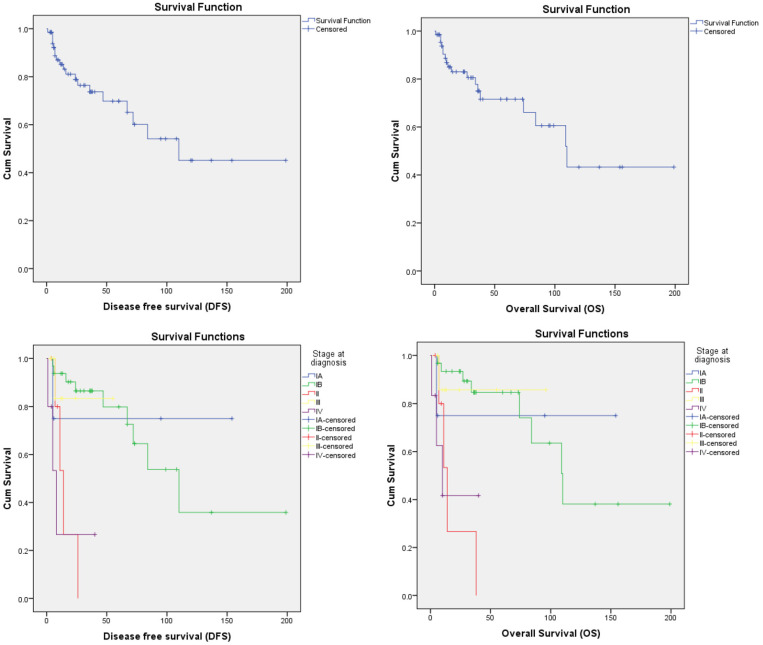
Kaplan–Meier curves for disease-free survival (DFS) (**left**) and overall survival (OS) (**right**) for the population study (**above**) and for the different stages of disease (**below**).

The outcomes following primary treatment were reported in 82% of the cases (*n* = 73). No evidence of disease (NED) was reported in 63%. On the other hand, 27.4% were dead of disease (DOD), while only 9.6% were alive with the disease (AWD). The 5-year disease-free survival and the 5-year overall survival were 65.2% and 66.1%, respectively. In the analysis, there were no statistically significant differences noted between the outcomes and the type of surgical management, the modality of adjuvant treatment, or the vaginal margin involvement. An advanced disease stage was identified as a predictor of inferior disease-free (<0.001) and overall survival (*p* < 0.001) (Figure A1).

## 4. Discussion

Mesonephric neoplasms originate from the epithelial remnants of the Wolffian (mesonephric) ducts. During early embryogenesis, around the fourth week of gestation, the mesonephric ducts connect the primitive kidney (mesonephros) to the cloaca, forming part of the male reproductive system [53]. In females, in the absence of anti-Müllerian hormone stimulation, the mesonephric ducts typically regress. However, in 1% to 20% of adults, and in up to 40% of children, remnants of these ducts may persist in the pelvis, often located in the lateral walls of the cervix, vagina, broad ligament, or near the ovarian hilum [29].

The majority of cervical cancers are strongly associated with human papillomavirus (HPV) infection, primarily presenting as squamous cell carcinoma (SCC), followed by adenocarcinoma [29]. HPV-induced cervical cancer is a prime example of how persistent viral infections can lead to malignancy. The oncogenic potential of HPV is due to its ability to transform host cells into immortalised cells, promoting uncontrolled proliferation and abnormal mitosis. Through microtrauma, HPV gains access to the basal layer of epithelial cells, where it replicates its DNA and alters the host cell’s tumour suppressor genes and cyclins to maintain cellular immortality. This leads to an accumulation of genetic mutations beyond repair, resulting in the characteristic excessive proliferation of the basal cell layer, a hallmark of HPV-induced cervical cancer [54].

In contrast to SCC, endocervical adenocarcinomas represent a heterogeneous group of cancers. Notably, 15–25% of all endocervical adenocarcinomas are HPV-independent and exhibit distinct molecular alterations [55].

**Figure 2:**
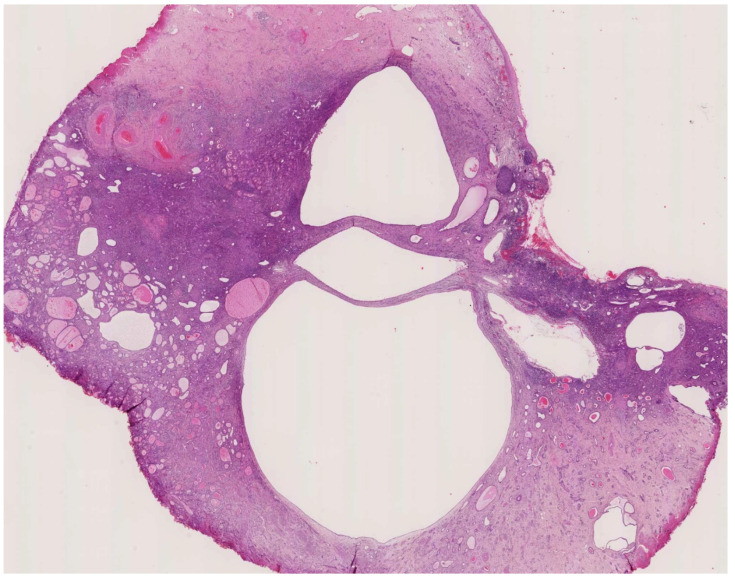
A low-power view of a mesonephric carcinoma of the cervix.

Mesonephric adenocarcinomas (MNACs) are rare non-mucinous neoplasms, constituting less than 1% of all cervical cancers. They most commonly arise from the lateral wall of the cervix, typically at the 3 and 9 o’clock positions [5,53]. A definitive diagnostic modality has not been established, but diagnostic approaches include Papanicolaou smears, cone biopsies, endometrial curettage, or an examination of hysterectomy specimens [4]. Evidence regarding the clinical course of MNAC is limited; however, common presentations include abnormal vaginal bleeding, adnexal masses upon pelvic examination, or incidental findings during routine check-ups [5]. Histologically, MNAC is highly infiltrative, displaying a variety of architectural patterns, such as tubular, solid, papillary, retiform, ductal (glandular), and sex-cord-like structures [5,53]. The tubular pattern may contain dense eosinophilic secretions, similar to that seen in mesonephric remnants and hyperplasia. The solid pattern may be partly spindled and contain heterologous elements (Figure 2). The presence of a solid growth pattern is associated with the worst prognosis [56,57]. Frequent mitotic figures can be noted. Histological variability often leads to the misclassification and underestimation of MNAC’s true incidence in the literature [12].

Immunohistochemically, MNACs typically exhibit luminal positivity with CD10 and diffuse positivity with CK7, PAX8, PAX2, epithelial membrane antigen (EMA), and vimentin [1,5,53]. They are consistently negative for CK20, oestrogen receptors (ERs), progesterone receptors (PRs), p16, and mCEA [7,31,53]. GATA3 is less positive compared to mesonephric remnants and hyperplasia [35,58]. However, the variability in staining patterns among mesonephric lesions has contributed to the absence of a definitive immunohistochemical profile for MNAC diagnosis. Positive staining for CD10, CK7, and calretinin, combined with negative immunostaining for CEA, strongly suggests a diagnosis of MNAC [5]. The immunohistochemical staining patterns of MNAC reported across the literature are summarised in Table 3.

MNACs are typically negative for HPV and frequently exhibit negative staining for p16, oestrogen receptors (ERs), and progesterone receptors (PRs). GATA3 and PAX8 are positive in over 85% of cases, while CD10 is present in just over 70% [58,59,60].

Clinically, MNACs are often aggressive and associated with poor outcomes, irrespective of the stage at presentation [5,7,12]. Our pooled analysis revealed that 37.9% of cases with available data experienced disease recurrence at a mean interval of 26.4 months, and 27.4% of patients died due to the disease regardless of the treatment modality.

As MNAC is an HPV-independent neoplasm, it follows a distinct pathophysiology and has been linked to several genetic mutations, including KRAS/NRAS, ARID1A, ARID1B, SMARCA4, and CTNNB1 mutations, along with chromosomal alterations such as gains of 1q, chromosomes 10 and 12, and loss of 1p [53,55,61]. Among these, KRAS/NRAS mutations are the most commonly associated with MNAC, being present in approximately 80% of cases. This genetic profile suggests the potential benefit of targeted therapies, such as KRAS/MAPK inhibitors, in treating MNAC [6]. Additionally, while around 14% of cervical adenocarcinomas may display HPV negativity for various reasons, MNAC is entirely distinct due to its unique molecular alterations compared to other HPV-negative cervical adenocarcinomas [6,55]. Notably, no microsatellite instability has been noted, but there are chromosomal abnormalities including gain of 1q, loss of 1p, and gain of chromosomes 10 and 12.

### 4.1. Chemotherapy and Radiotherapy Considerations

Cervical cancer is treated on the basis of HPV-related neoplasms employing either radical surgery for early-stage tumours less than 4 cm or chemoradiotherapy for locally advanced and distantly metastasising cancer [62,63]. The most common chemotherapeutic regimen for cervical cancer is platinum-based treatment with paclitaxel added for cases with distant metastasis [62]. Cases associated with a high risk of recurrence, due to positive margins, positive lymph nodes, or LVSI-positive tumours, are considered for adjuvant chemotherapy. The predominant chemotherapeutic regimen among reported cases of MNAC included platinum-based chemotherapy (cisplatin or carboplatin) with paclitaxel [12,14,16,36,64]. Such combination has shown expected popularity as it is effective against locally advanced cervical cancer, easily administered, and associated with low toxicity [14].

Based on the high rate of recurrence reported in our review (37.9%), in patients receiving chemotherapy, one could conclude that the currently used chemotherapeutic regimens in advanced MNACs are ineffective. This hypothesis could further be supported by the absence of clear evidence on the biological behaviour of MNAC and the effectiveness of chemotherapy in overall and disease-free survival [10,12,31,65]. Moreover, a reliable evaluation of the survival outcomes of MNAC cases receiving common treatment regimens is not feasible due to the disease’s rarity. Since chemotherapy and its targets are designed on a biological basis, the use of chemotherapeutic agents designed to treat HPV-related cervical cancer may not be beneficial in MNAC, as the basic underlying pathophysiology is completely different between HPV- and non-HPV-related tumours. As noted by Praiss et al., the frequent recurrence of MNAC and its tendency to metastasize to the lungs highlight the potential importance of targeting specific molecular pathways, such as the MAPK pathway [66]. A prospective single-institution study (NCT05787561) is currently investigating the efficacy of targeted therapy in this context using a combination of the MEK/RAF inhibitor avutometinib and the FAK inhibitor defactinib in patients with recurrent mesonephric adenocarcinoma (MA) or mesonephric-like adenocarcinoma (MLA) of gynaecologic origin [66,67].

Knowledge of the embryological origin of MNAC should prompt treatment similar to that effectively used for other mesonephric cancers. We suggest that consideration should be given to a regimen comprising vincristine and dactinomycin, with or without doxorubicin, which is the most effective chemotherapeutic regimen utilised in mesonephric tumours of the kidney (e.g., nephroblastoma) [68]. Wilms’ tumour (or nephroblastoma) is the most common renal paediatric malignancy [68]. In the same fashion as MNAC, this tumour originates from the intermediate mesoderm and is composed of different blastemal, stromal, and epithelial elements [69,70]. Chemotherapy in favourable histological variants of Wilms’ tumour results in an overall survival rate of more than 90% [68]. Recently, Montalvo et al. (2019) documented the CTNNB1 mutations in a case of MNAC [5]. This discovery could elucidate the link between Wilms’ tumour and MNAC as it is already known that CTNNB1 is the most commonly mutated gene in Wilms’ tumour [71]. This mutation is a Wnt activating alteration which, in turn, stabilises beta catenin-1 that further induces tumour cell proliferation and inhibits proper cellular adhesion [5,71]. Nonetheless, irrespective of the origin and biological behaviour of MNAC, surgical resection is the primary and most effective therapeutic modality.

### 4.2. Surgical Considerations Based on Embryology and Anatomical Relations

The intermediate mesoderm is the primary germ cell layer from which both the kidney and reproductive system originate, giving rise to the pronephros, mesonephros, and metanephros. In early female embryological development, the paramesonephric ducts (Müllerian ducts) fuse with the mesonephric ducts (Wolffian ducts) to form the uterovaginal canal, which eventually develops into the uterine tubes, uterus, cervix, and the upper third of the vagina. The paramesonephric ducts, upon contact with the urogenital sinus, induce the formation of the sinus tubercle. This interaction stimulates the development of two sinovaginal bulbs, which fuse to form the vaginal plate. As the vaginal plate grows cranially, it separates the urogenital sinus from the uterus, forming the lower vagina, which is ultimately separated from the sinus by the hymen [72].

Alternatively, it has been hypothesised that the vagina may arise from the downward growth of both mesonephric and paramesonephric ducts, suggesting that the vaginal plate is derived from the caudal segments of the mesonephric duct. During the 10th week of development, the mesonephric ducts regress. However, remnants may persist, as noted by Huffman in 1948, within the lateral uterine wall, contributing to structures like the epoophoron and paroophoron [2]. These remnants, located within the middle layer of the cervix, may extend into the vagina and terminate near the hymen (Figure 2). The mesonephric remnants acquire stromal and muscular components from the cervix, contributing to their persistence [2].

Both the paramesonephric and mesonephric ducts share similarities in their biological composition. Initially positioned medially, the mesonephric ducts are later crossed ventrally by the descending paramesonephric ducts, which fuse near the urogenital sinus, forming the Müllerian tubercle. The mesonephric ducts migrate medially along the dorsal wall of the sinus as they separate from the ureters. This relationship influences the development of the uterovaginal canal and its connections [72,73].

Radical hysterectomy, as described in surgical techniques such as Wertheim’s, Meigs’, and the more recent Querleu–Morrow classification, remains the mainstay of treatment for cervical cancer [74,75]. These procedures aim to achieve tumour-free margins, remove metastatic lymph nodes, and provide adjuvant treatment [76]. However, Querleu et al. emphasised the importance of tailoring the radicality of the procedure based on individual patient factors, including surgical margins and risk of lymphatic spread [75]. Despite the refined surgical techniques, no clear evidence supports a specific extent of radical hysterectomy for mesonephric adenocarcinoma (MNAC).

Given the embryological origins of MNAC, which arise from mesonephric ducts lateral to their Müllerian counterparts, it is logical to consider modifying surgical approaches for MNAC. Unlike typical cervical cancers, MNACs may extend beyond the cervix into the upper vagina, warranting more extensive vaginal resection. Current practice typically limits resection to 10 mm of the vagina, as MNACs are often managed similarly to cervical cancers. However, this approach may not be appropriate for MNAC, as it is a malignant growth of mesonephric origin rather than a true cervical cancer. Therefore, resection should consider the potential spread of MNAC into the upper vagina, reflecting its embryological origins.

We propose that a surgical modification involving resection of a more substantial portion of the upper vagina, specifically up to one-third of its length (approximately 3 cm from the cervix), might be considered for mesonephric adenocarcinoma of the uterine cervix. This is based on the embryological origins of MNAC, which differ from typical cervical cancers and may warrant a more tailored surgical approach. This approach aligns with Muallem’s technique, which emphasises the importance of resecting the vaginal cuff during radical hysterectomy in cervical cancers [77].

**Figure 3:**
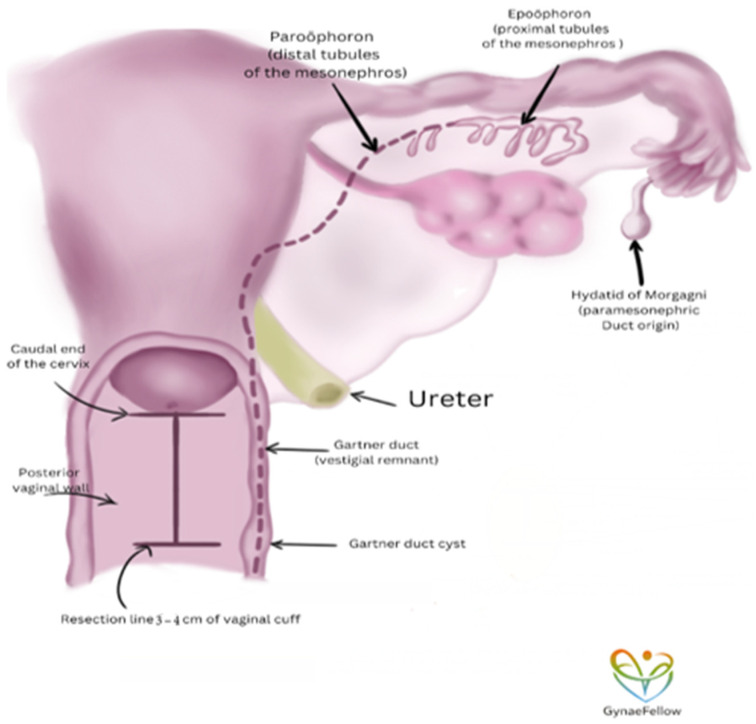
Proposed surgical approach with embryology and anatomical relations. Figure depicts proposed extensive vertical resection of vaginal vault (2–4 cm) to ensure clear, negative margins. Figure courtesy of https://play.google.com/store/apps/details?id=com.gynaefellow.platform&pli=1 accessed on 20 June 2022.

Muallem’s classification of radical hysterectomy highlights the need for a vertical radical approach, addressing the three-dimensional parametrium and paracolpium [77]. While this approach differs from the conventional horizontal resection used in standard radical hysterectomies, as the upper third of the vagina is significant for both vascular and lymphatic connections to the cervix and uterus, it could offer theoretical benefits for achieving clear surgical margins and addressing the anatomical considerations unique to MNAC. We propose implementing a modified Type III radical hysterectomy, similar to Class C1 in the Querleu–Morrow classification, which involves complete resection of the dorsal, ventral, and lateral parametrium along with extensive vertical resection of the vaginal vault (2–4 cm) to ensure clear, negative margins and minimise the need for adjuvant chemotherapy or radiotherapy. This approach correlates with the anatomical location of mesonephric duct remnants and is likely to improve surgical outcomes for patients with MNAC (Figure 3).

### 4.3. Strengths and Limitations of Our Review

This study highlights the need for a structured management plan for mesonephric adenocarcinoma (MNAC), potentially revisiting the embryological origine of the tumour to refine a potentially more effective tailored surgical approach. Further research could clarify the benefits of individualised surgical and adjunct therapies.

Given the rarity of mesonephric cervical carcinoma, our review is limited by reliance on case reports and case series, including potential misdiagnosis due to the variability of diagnostic criteria that might have been used, as we did not independently verify diagnoses or set standardised diagnostic criteria.

## 5. Conclusions

Mesonephric neoplasms of the uterine cervix are rare and clinically aggressive cancers that indicate a poor prognosis. Accurate identification and effective management can be challenging due to their particular anatomic and immunohistochemical characteristics. Therefore, a more tailored embryological-based approach should be considered for an optimal oncologic outcome. Future studies would be instrumental in determining the precise role and benefits of such an approach for optimising outcomes in patients with this rare malignancy.

## Figures and Tables

**Table 1:** The baseline clinical characteristics, treatment modalities, and outcomes reported in the included studies.

Study	Year	Case	Stage	Type of Treatment	Recurrence	DFS	Outcome	Follow Up Time	OS
Tan et al. [18]	2024	1	N/A	RAH, BSO, PLND, SLND	no	15	NED	15	15
		2	N/A	TAH, BSO, PLND, O, CS, AP	no	5	NED	5	5
		3	N/A	RAH, BSO, PLND, PALND, O, AP	no	3	NED	3	3
Kuratsune et al. [19]	2024	4	IIA2	RAH, BSO, PLND	no	13	NED	13	13
Kobayashi et al. [20]	2024	5	IIB	RT	lungs	4	NED	96	96
Li et al. [21]	2023	6	N/A	TAH, BSO, PLND	no	-	N/A	N/A	N/A
Devarashetty et al. [22]	2022	7	IB	RAH	lungs	36	AWD	36	36
Kumar et al. [23]	2022	8	N/A	Wide margin vaginal vault excision	N/A	N/A	NED	N/A	N/A
Xie et al. [24]	2021	9	IB1	RAH, BSO, PLND	N/A	N/A	N/A	64	64
		10	IB1	RAH, BSO, PLND	N/A	N/A	N/A	70	70
Nili et al. [25]	2021	11	IB2	TAH, BSO	pelvic peritoneal metastasis, liver, lungs	9	AWD	9	9
Reis-de-Carvalho et al. [26]	2021	12	IB1	RAH (Type C Q-M), Vaginal margin 22 mm, BSO, PLND	N/A	60	NED	60	60
Dinh et al. [27]	2021	14	IIIC1	Robotically assisted TLH, BSO, PLND	no	-	NED	N/A	N/A
Jiang et al. [28]	2020	13	N/A	TAH, BSO, PLND, O, AP	lungs	32	AWD	32	32
Papoutsis et al. [15]	2019	15	IIB	RAH, BSO, PLND	no	12	NED	12	12
Montalvo et al. [5]	2019	16	N/A	TAH	lungs	36	N/A	36	36
Ribeiro et al. [16]	2019	17	IIA	RAH, BSO, PLND, O	bone	7	DOD	7	7
Cavalcanti et al. [29]	2017	18	IB	RAH, BSO, PLND	pelvis	14	DOD	14	14
Kır et al. [30]	2016	19	N/A	RAH, BSO, PLND	N/A	N/A	N/A	N/A	N/A
Puljiz et al. [31]	2016	20	IB	RAH, BSO, PLND, PALND	no	36	NED	36	36
Ditto et al. [32]	2016	21	IIB	RAH, BSO, PLND	no	6	NED	6	6
Dierickx et al. [12]	2016	22	IIB	RAH (Type 2 Wertheim), BSO	no	24	NED	24	24
Yeo et al. [33]	2016	23	IVB	CT	lungs	N/A	N/A	N/A	N/A
Tekin et al. [34]	2015	24	1B2	TAH, BSO, PLND, PALND	no	N/A	N/A	N/A	N/A
Mirkovic et al. [6]	2015	25	IB	N/A	no	67	NED	67	67
		26	IIIB	N/A	abdomen, pelvis	N/A	AWD	10	10
		27	N/A	N/A	no	-	N/A	N/A	N/A
Roma et al. [35]	2014	28	I	TAH, BSO	no	-	N/A	N/A	N/A
Tseng et al. [36]	2014	29	IIIB	TAH, BSO, PLND, O	no	4	NED	4	4
Abdul-Ghafar et al. [3]	2013	30	N/A	Vaginal hysterectomy	No	24	NED	24	24
Menon et al. [37]	2013	31	IB	TAH, BSO, PLND	no	6	NED	6	6
Meguro et al. [38]	2013	32	IIA	RAH, BSO, PLND	local	7	NED	10	10
Lopez-Chardi et al. [39]	2013	74	IB	TAH, BSO	no	31	NED	31	31
Nomoto et al. [40]	2012	33	IB	TAH, BSO, PLND	lungs	12	N/A	12	12
		34	IB	TAH, BSO, PLND	no	N/A	N/A	N/A	N/A
Anagnostopoulos et al. [4]	2012	35	IB	RAH, BSO, PLND	no	6	NED	6	6
Fukunaga et al. [41]	2008	36	IB	TAH, BSO, PLND, O	no	4	NED	4	4
Yap et al. [7]	2006	37	IB	RAH, BSO, PLND, PALND	no	37	NED	37	37
Bagué et al. [10]	2004	38	IB	TAH, BSO, PLND	no	137	NED	137	137
		39	IIA	N/A	no	N/A	N/A	N/A	N/A
		40	IB	TAH, USO	no	37	NED	37	37
		41	IIA	TAH, BSO, PLND, O	yes—not specified	7	DOD	7	7
		42	IVB	TAH, BSO	bone	40	AWD	40	40
		43	IB	TAH, BSO, PLND, O	no	13	NED	13	13
Angeles et al. [42]	2004	44	N/A	TAH	no	12	NED	12	12
McNall et al. [14]	2004	45	III	Resection of mass with partial resection of posterior vaginal wall, PLND, BSO	no	55	NED	55	55
Silver et al. [13]	2001	46	IB	TAH, USO	no	18	NED	18	18
		47	IB	TAH, BSO	no	25	NED	25	25
		48	IB	TAH, BSO	no	38	NED	38	38
		49	IB	TAH, BSO, PLND	no	73	NED	73	73
		50	IB	TAH, BSO	no	99	NED	99	99
		51	IB	TAH, BSO, PLND	rectovaginal	20	NED	30	30
		52	IB	TAH, BSO	mediastinum	67	DOD	74	74
		53	IB	TAH, BSO, PLND	no	N/A	N/A	N/A	N/A
		54	N/A	TAH, BSO	no	38	NED	89	89
		55	IIB	Biopsy	pelvis	26	DOD	38	38
		56	IVB	TAH, BSO, PLND, O	bladder	8	DOD	10	10
Clement et al. [9]	1995	57	IB	TAH, BSO, PLND	no	24	NED	24	24
		58	IB	TAH, BSO, PLND	abdomen	12	NED	24	24
		59	IB	TAH, BSO, PLND	abdomen	5	DOD	9	9
		60	IB	TAH, BSO, PLND	no	N/A	N/A	N/A	N/A
		61	IB	TAH, BSO, PLND	abdomen	108	AWD	156	156
		62	IB	TAH, BSO	no	36	NED	36	36
		63	IB	TAH, BSO	no	28	NED	28	28
		64	IB	TAH, BSO	no	N/A	N/A	N/A	N/A
Stewart et al. [43]	1993	65	N/A	RAH, BSO, PLND	no	120	NED	120	120
Ferry et al. [8]	1990	66	N/A	TAH, BSO	no	60	NED	60	60
Lang et al. [44]	1990	67	N/A	TAH	no	10	NED	10	10
		68	IB	TAH	no	N/A	N/A	N/A	N/A
Valente et al. [45]	1987	69	IB	RAH, BSO, PLND	pelvis	24	DOD	34	34
Buntine et al. [46]	1979	70	IB	TAH, BSO	vagina	84	DOD	109	109
Rosen et al. [47]	1975	75	1B	RAH, PLND, AP	cervix	16	DOD	27	27
Hart, Norris et al. [48]	1972	76	I	TAH, BSO	no	73	NED	73	73
		77	I	TAH, BSO, PLND	no	199	NED	199	199
		78	I	TAH, BSO, PLND	no	N/a	NED	N/A	N/A
		79	I	TAH, BSO, PLND	no	154	NED	154	154
		80	I	TAH, BSO	no	95	NED	95	95
		81	I	TAH, BSO, PLND	no	5	DOD	5	5
		82	I	RT	no	110	DOD	110	110
		83	I	Cobalt	no	6	DOD	6	6
		84	I	Local Excision	no	47	DOD	47	47
		85	II	RT	no	11	DOD	11	11
		86	IV	X-ray	no	5	DOD	5	5
		87	IV	X-ray	no	1	DOD	1	1
		88	I	TAH, USO	no	N/A	N/A	N/A	N/A
Tóth et al. [49]	1964	89	N/A	TAH, BSO, upper one-third vaginectomy	no	121	NED	12	2
		90	N/A	Curettage	no	-	DOD	N/A	N/A
		91	III	RT	yes—not specified	3	AWD	N/A	N/A
Zaczek et al. [50]	1963	71	N/A	TAH	pelvis	36	DOD	36	36
McGee et al. [51]	1962	72	IB	TAH	pelvis	72	DOD	84	84
Rose et al. [52]	1960	73	N/A	N/A	no	N/A	N/A	N/A	N/A

RAH: radical abdominal hysterectomy, TAH: total abdominal hysterectomy, BSO: bilateral salpingo-oophorectomy, USO: unilateral salpingo-oophorectomy, PLND: pelvic lymphadenectomy, O: omentectomy, AP: appendectomy, Q-M: Querleu-Morrow, CT: chemotherapy, RT: radiotherapy. NED: no evidence of disease. AWD: alive with disease. DOD: dead of disease. N/A: not available.

**Table 2:** Patient characteristics, treatment details, and follow-up outcomes.

Descriptives	Value	
	Mean Value/Number	Range/Percentage
1. Age (years)	50.86 ± 13.8	13–77
2. Stage		
I	51	70.8%
II	11	15.4%
III	5	6.9%
IV	5	6.9%
3. Hysterectomy		
RAH	21	23.1%
TAH	56	61.5%
No hysterectomy	14	15.4%
4. BSO/USO		
yes	70	76.9%
no	7	7.7%
n/a	14	15.4%
5. PLND		
yes	46	50.5%
no	40	44%
n/a	5	5.5%
6. Vaginal margin (data available for 21 cases)		
positive	10	47.6%
negative	11	52.4%
n/a	70	76.9%
7. Type of adjuvant treatment (data available for 45 cases)		
CT	7	15.6%
RT	24	53.3%
CRT	14	31.1%
n/a	46	50.5%
8. Follow-up (months)	29	1–199
9. Recurrence (data available for 71 cases)		
yes	25	35.2%
no	46	64.8%
n/a	20	27.5%
10. Death (data available for 73 cases)		
yes	20	27.4%
no	53	72.6%
n/a	18	19.8%

RAH: radical abdominal hysterectomy, TAH: total abdominal hysterectomy, BSO: bilateral salpingo-oophorectomy, USO: unilateral salpingo-oophorectomy, PLND: pelvic lymphadenectomy, CT: chemotherapy, RT: radiotherapy, CRT: chemoradiotherapy, n/a: not available.

**Table 3:** The immunohistochemical staining patterns of MNAC reported across the included studies.

Marker	Status
Epithelial Membrane Antigen (EMA)	Consistently positive
CD10	Consistently positive
Cytokeratin (CK) *	Consistently positive
Cytokeratin (CAM 5.2)	Consistently positive
GATA3	Consistently positive
PAX2, PAX8	Consistently positive
Oestrogen (ER) and Progesterone Receptors (PR)	Consistently negative
Monoclonal Carcinoembyronic Antigen (mCEA)	Consistently negative
p16	Consistently negative
Calretinin and Vimentin	Variable **
p53	Consistently positive
Wilms Tumour—1 (WT-1)	Variable
Cytokeratin (CK20)	Consistently negative
Inhibin	Variable
Synaptophysin and Chromogranin	Consistently negative or weakly positive
PTEN	Positive
CD1	Negative
Actin—M851	Negative
E—cadherin	Positive
CK18 and CD56	Negative
TAG—72	Positive
Cytokeratin (CK 13, 14, and 16)	Variable
S100 Protein	Positive
Gross Cystic Disease Fluid Protein (GCDFP)	Negative

Status can be consistently positive, consistently negative, or variable. * Most commonly CK7. ** Variable with trend towards positivity.

## Data Availability

The datasets used in this study can be found in the full-text articles included in the systematic review.

## Appendix A

**Table A1:** Risk of bias quality assessment based on JBI critical appraisal checklists for case series and case reports.

Study	D1 *	D2	D3	D4	D5	D6	D7	D8	D9	D10	Overall RoB **
Tan et al. [18]	yes	yes	yes	yes	yes	yes	yes	yes	unclear	unclear	Low
Kuratsune et al. [19]	yes	yes	yes	yes	yes	yes	no	yes	N/A	N/A	Low
Kobayashi et al. [20]	yes	yes	yes	yes	yes	yes	yes	yes	N/A	N/A	Low
Li et al. [21]	yes	yes	yes	yes	yes	yes	no	yes	N/A	N/A	Low
Devarashetty et al. [22]	yes	yes	yes	yes	yes	yes	no	yes	N/A	N/A	Low
Kumar et al. [23]	yes	yes	yes	yes	yes	yes	no	yes	N/A	N/A	Low
Xie et al. [24]	yes	yes	yes	yes	yes	yes	yes	yes	unclear	yes	Low
Nili et al. [25]	yes	yes	yes	yes	yes	yes	yes	yes	N/A	N/A	Low
Reis-de-Carvalho et al. [26]	yes	yes	yes	yes	yes	yes	no	yes	N/A	N/A	Low
Jiang et al. [28]	yes	yes	yes	yes	yes	yes	yes	yes	N/A	N/A	Low
Dinh et al. [27]	yes	yes	yes	yes	yes	yes	yes	yes	N/A	N/A	Low
Papoutsis et al. [15]	yes	yes	yes	yes	yes	yes	no	yes	N/A	N/A	Low
Montalvo et al. [5]	yes	yes	yes	yes	yes	yes	no	yes	N/A	N/A	Low
Ribeiro et al. [16]	yes	yes	yes	yes	yes	yes	yes	yes	N/A	N/A	Low
Cavalcanti et al. [29]	yes	yes	yes	yes	yes	yes	yes	yes	N/A	N/A	Low
Kır et al. [30]	yes	yes	yes	no	yes	no	no	yes	N/A	N/A	High
Puljiz et al. [31]	yes	yes	yes	yes	yes	yes	no	yes	N/A	N/A	Low
Ditto et al. [32]	yes	yes	yes	yes	yes	yes	yes	yes	N/A	N/A	Low
Dierickx et al. [12]	yes	yes	yes	yes	yes	yes	no	yes	N/A	N/A	Low
Yeo et al. [33]	yes	yes	yes	yes	no	no	no	yes	N/A	N/A	High
Tekin et al. [34]	yes	yes	yes	yes	yes	no	no	yes	N/A	N/A	Moderate
Mirkovic et al. [6]	yes	yes	yes	no	yes	yes	yes	yes	unclear	yes	Moderate
Roma et al. [35]	yes	yes	yes	yes	yes	no	no	yes	N/A	N/A	Moderate
Tseng et al. [36]	yes	yes	yes	yes	yes	yes	yes	yes	N/A	N/A	Low
Abdul-Ghafar et al. [3]	yes	yes	yes	yes	yes	yes	no	yes	N/A	N/A	Low
Menon et al. [37]	yes	yes	yes	yes	yes	yes	no	yes	N/A	N/A	Low
Meguro et al. [38]	yes	yes	yes	yes	yes	yes	yes	yes	N/A	N/A	Low
Nomoto et al. [40]	yes	yes	yes	yes	yes	no	no	yes	N/A	N/A	High
Anagnostopoulos et al. [4]	yes	yes	yes	yes	yes	yes	no	yes	N/A	N/A	Low
Fukunaga et al. [41]	yes	yes	yes	yes	yes	no	no	yes	N/A	N/A	Moderate
Yap et al. [7]	yes	yes	yes	yes	yes	yes	no	yes	N/A	N/A	Low
Bagué et al. [10]	yes	yes	yes	no	yes	yes	yes	unclear	no	no	Moderate
Angeles et al. [42]	yes	yes	yes	yes	yes	yes	no	yes	N/A	N/A	Low
McNall et al. [14]	yes	yes	yes	yes	yes	yes	no	yes	N/A	N/A	Low
Silver et al. [13]	yes	yes	yes	yes	yes	yes	yes	unclear	no	no	Moderate
Clement et al. [9]	yes	yes	yes	no	yes	yes	yes	yes	unclear	no	Moderate
Stewart et al. [43]	yes	yes	yes	no	yes	no	no	yes	N/A	N/A	Moderate
Ferry et al. [8]	yes	yes	no	no	yes	no	no	yes	N/A	N/A	High
Lang et al. [44]	yes	yes	no	no	yes	no	no	yes	N/A	N/A	High
Valente et al. [45]	yes	yes	yes	yes	yes	yes	yes	yes	N/A	N/A	Low
Buntine et al. [46]	yes	yes	yes	yes	yes	yes	yes	yes	N/A	N/A	Low
Zaczek et al. [50]	yes	yes	yes	yes	yes	yes	yes	yes	N/A	N/A	Low
McGee et al. [51]	yes	yes	yes	yes	yes	yes	yes	yes	N/A	N/A	Low
Rose et al. [52]	yes	yes	yes	no	no	no	no	no	N/A	N/A	High
Lopez-Chardi et al. [39]	yes	yes	yes	yes	yes	yes	yes	yes	N/A	N/A	Low
Rosen et al. [47]	yes	yes	yes	yes	yes	yes	yes	yes	N/A	N/A	Low
Hart, Norris et al. [48]	yes	yes	yes	yes	yes	yes	yes	yes	unclear	yes	Low
Tóth et al. [49]	yes	yes	yes	yes	yes	yes	yes	yes	N/A	N/A	Low

JBI: Joanna Briggs Institute. * D1–D10 indicate the domains assessed based on the JBI Critical Appraisal Checklists for case reports and case series. ** The risk of bias was classified as high when the study reached up to 49% of “yes” scores, moderate when the study reached from 50 to 69% of “yes” scores, and low when the study reached more than 70% of “yes” scores.

## References

[1] Stolnicu S., Barsan I., Hoang L., Patel P., Terinte C., Pesci A., Aviel-Ronen S., Kiyokawa T., Alvarado-Cabrero I., Pike M.C. (2018). International Endocervical Adenocarcinoma Criteria and Classification (IECC): A New Pathogenetic Classification for Invasive Adenocarcinomas of the Endocervix. Am. J. Surg. Pathol..

[2] Huffman J.W. (1948). Mesonephric Remnants in the Cervix. Am. J. Obstet. Gynecol..

[3] Abdul-Ghafar J., Chong Y., Han H., Cha D., Eom M. (2013). Mesonephric Adenocarcinoma of the Uterine Cervix Associated with Florid Mesonephric Hyperplasia: A Case Report. J. Lifestyle Med..

[4] Anagnostopoulos A., Ruthven S., Kingston R. (2012). Mesonephric Adenocarcinoma of the Uterine Cervix and Literature Review. BMJ Case Rep..

[5] Montalvo N., Redrobán L., Galarza D. (2019). Mesonephric Adenocarcinoma of the Cervix: A Case Report with a Three-Year Follow-Up, Lung Metastases, and Next-Generation Sequencing Analysis. Diagn. Pathol..

[6] Mirkovic J., Sholl L.M., Garcia E., Lindeman N., MacConaill L., Hirsch M., Dal Cin P., Gorman M., Barletta J.A., Nucci M.R. (2015). Targeted Genomic Profiling Reveals Recurrent KRAS Mutations and Gain of Chromosome 1q in Mesonephric Carcinomas of the Female Genital Tract. Mod. Pathol..

[7] Yap O.W.S., Hendrickson M.R., Teng N.N.H., Kapp D.S. (2006). Mesonephric Adenocarcinoma of the Cervix: A Case Report and Review of the Literature. Gynecol. Oncol..

[8] Ferry J.A., Scully R.E. (1990). Mesonephric Remnants, Hyperplasia, and Neoplasia in the Uterine Cervix. A Study of 49 Cases. Am. J. Surg. Pathol..

[9] Clement P.B., Young R.H., Keh P., Ostör A.G., Scully R.E. (1995). Malignant Mesonephric Neoplasms of the Uterine Cervix. A Report of Eight Cases, Including Four with a Malignant Spindle Cell Component. Am. J. Surg. Pathol..

[10] Bagué S., Rodríguez I.M., Prat J. (2004). Malignant Mesonephric Tumors of the Female Genital Tract: A Clinicopathologic Study of 9 Cases. Am. J. Surg. Pathol..

[11] Cina S.J., Richardson M.S., Austin R.M., Kurman R.J. (1997). Immunohistochemical Staining for Ki-67 Antigen, Carcinoembryonic Antigen, and P53 in the Differential Diagnosis of Glandular Lesions of the Cervix. Mod. Pathol..

[12] Dierickx A., Göker M., Braems G., Tummers P., Van den Broecke R. (2016). Mesonephric Adenocarcinoma of the Cervix: Case Report and Literature Review. Gynecol. Oncol. Rep..

[13] Silver S.A., Devouassoux-Shisheboran M., Mezzetti T.P., Tavassoli F.A. (2001). Mesonephric Adenocarcinomas of the Uterine Cervix: A Study of 11 Cases with Immunohistochemical Findings. Am. J. Surg. Pathol..

[14] McNall R.Y., Nowicki P.D., Miller B., Billups C.A., Liu T., Daw N.C. (2004). Adenocarcinoma of the Cervix and Vagina in Pediatric Patients. Pediatr. Blood Cancer.

[15] Papoutsis D., Sahu B., Kelly J., Antonakou A. (2019). Perivascular Epithelioid Cell Tumour and Mesonephric Adenocarcinoma of the Uterine Cervix: An Unknown Co-Existence. Oxf. Med. Case Rep..

[16] Ribeiro B., Silva R., Dias R., Patrício V. (2019). Carcinosarcoma of the Uterine Cervix: A Rare Pathological Finding Originating from Mesonephric Remnants. BMJ Case Rep..

[17] Page M.J., Moher D., Bossuyt P.M., Boutron I., Hoffmann T.C., Mulrow C.D., Shamseer L., Tetzlaff J.M., Akl E.A., Brennan S.E. (2021). PRISMA 2020 Explanation and Elaboration: Updated Guidance and Exemplars for Reporting Systematic Reviews. BMJ.

[18] Tan C.-Y., Li T.-T., Xu N., Guo H.-H. (2024). Mesonephric Adenocarcinoma of the Cervix: An Analysis for 3 Cases and Literature Review. Asian J. Surg..

[19] Kuratsune K., Ueda T., Tajiri R., Tohyama A., Hoshino K., Harada H., Kurita T., Kubo C., Komatsu K., Shiba E. (2024). A Case of Adenocarcinoma, HPV-Independent, Mesonephric Type with Significant Response to Neoadjuvant Chemotherapy. J. UOEH.

[20] Kobayashi N., Oike T., Ando K., Murata K., Tamaki T., Noda S.-E., Kogure K., Nobusawa S., Oyama T., Ohno T. (2024). Carbon Ion Radiotherapy for Mesonephric Adenocarcinoma of the Uterine Cervix: A Case Report. J. Med. Case Rep..

[21] Li X., Liu H., Zhang Y., Liu Y. (2023). A Rare Case of Mesonephric Adenocarcinoma in the Uterine Cervix. Asian J. Surg..

[22] Devarashetty S., Chennapragada S.S., Mansour R. (2022). Not Your Typical Adenocarcinoma: A Case of Mesonephric Adenocarcinoma of the Cervix with Fibroblast Growth Factor Receptor 2 (FGFR2) Mutation. Cureus.

[23] Kumar S., Saklani B., Kapil R., Sen R. (2022). Post Hysterectomy Mesonephric Carcinoma: A Case Report and Literature Review. J. Cancer Res. Ther..

[24] Xie C., Chen Q., Shen Y. (2021). Mesonephric Adenocarcinomas in Female Genital Tract: A Case Series. Medicine.

[25] Nili F., Salarvand S., Saffar H., Kalaghchi B., Ghalehtaki R. (2021). Mesonephric Adenocarcinoma of Uterine Cervix; A Case Report and Review of the Literature. Iran. J. Pathol..

[26] Reis-de-Carvalho C., Vaz-de-Macedo C., Ortiz S., Colaço A., Calhaz-Jorge C. (2021). Cervical Mesonephric Adenocarcinoma: A Case Report of a Rare Gynecological Tumor from Embryological Remains of the Female Genital Tract. Rev. Bras. Ginecol. Obstet..

[27] Dinh T.-K.T., Parker E.U., Gangadhar K., Mansoori B., Dyer B.A. (2021). Management of Locally Advanced Mesonephric Carcinoma of the Cervix in the Setting of Mullerian Duct Anomaly Spectrum and Unilateral Renal Agenesis: A Case Report and Review of the Literature. Brachytherapy.

[28] Jiang L.-L., Tong D.-M., Feng Z.-Y., Liu K.-R. (2020). Mesonephric Adenocarcinoma of the Uterine Cervix with Rare Lung Metastases: A Case Report and Review of the Literature. World J. Clin. Cases.

[29] Cavalcanti M.S., Schultheis A.M., Ho C., Wang L., DeLair D.F., Weigelt B., Gardner G., Lichtman S.M., Hameed M., Park K.J. (2017). Mixed Mesonephric Adenocarcinoma and High-Grade Neuroendocrine Carcinoma of the Uterine Cervix: Case Description of a Previously Unreported Entity with Insights into Its Molecular Pathogenesis. Int. J. Gynecol. Pathol..

[30] Kır G., Seneldir H., Kıran G. (2016). A Case of Mesonephric Adenocarcinoma of the Uterine Cervix Mimicking an Endometrial Clear Cell Carcinoma in the Curettage Specimen. J. Obstet. Gynaecol..

[31] Puljiz M., Danolić D., Kostić L., Alvir I., Tomica D., Mamić I., Munivrana I.V., Puljiz M., Vrdoljak D.V., Balja M.P. (2016). Mesonephric Adenocarcinoma of Endocervix with Lobular Mesonephric Hyperplasia: Case Report. Acta Clin. Croat..

[32] Ditto A., Martinelli F., Bogani G., Gasparri M.L., Donato V.D., Paolini B., Carcangiu M.L., Lorusso D., Raspagliesi F. (2016). Bulky Mesonephric Adenocarcinoma of the Uterine Cervix Treated with Neoadjuvant Chemotherapy and Radical Surgery: Report of the First Case. Tumori. J..

[33] Yeo M.K., Choi S.Y., Kim M., Kim K.H., Suh K.S. (2016). Malignant Mesonephric Tumor of the Cervix with an Initial Manifestation as Pulmonary Metastasis: Case Report and Review of the Literature. Eur. J. Gynaecol. Oncol..

[34] Tekin L., Yazici A., Akbaba E., Akin M.N. (2015). Mesonephric Adenocarcinoma of the Uterine Cervix: A Case Report and Review of the Literature. J. Pak. Med. Assoc..

[35] Roma A.A. (2014). Mesonephric Carcinosarcoma Involving Uterine Cervix and Vagina: Report of 2 Cases with Immunohistochemical Positivity for PAX2, PAX8, and GATA-3. Int. J. Gynecol. Pathol..

[36] Tseng C.-E., Chen C.-H., Chen S.-J., Chi C.-L. (2014). Tumor Rupture as an Initial Manifestation of Malignant Mesonephric Mixed Tumor: A Case Report and Review of the Literature. Int. J. Clin. Exp. Pathol..

[37] Menon S., Kathuria K., Deodhar K., Kerkar R. (2013). Mesonephric Adenocarcinoma (Endometrioid Type) of Endocervix with Diffuse Mesonephric Hyperplasia Involving Cervical Wall and Myometrium: An Unusual Case Report. Indian J. Pathol. Microbiol..

[38] Meguro S., Yasuda M., Shimizu M., Kurosaki A., Fujiwara K. (2013). Mesonephric Adenocarcinoma with a Sarcomatous Component, a Notable Subtype of Cervical Carcinosarcoma: A Case Report and Review of the Literature. Diagn. Pathol..

[39] Lopez-Chardi L., González-Bosquet E., Rovira Zurriaga C., Laïlla Vicens J.M. (2013). Mesonephric Carcinosarcoma of the Uterine Cervix: A Case Report. Eur. J. Gynaecol. Oncol..

[40] Nomoto K., Hayashi S., Tsuneyama K., Hori T., Ishizawa S. (2013). Cytopathology of Cervical Mesonephric Adenocarcinoma: A Report of Two Cases. Cytopathology.

[41] Fukunaga M., Takahashi H., Yasuda M. (2008). Mesonephric Adenocarcinoma of the Uterine Cervix: A Case Report with Immunohistochemical and Ultrastructural Studies. Pathol. Res. Pract..

[42] Angeles R.M., August C.Z., Weisenberg E. (2004). Pathologic Quiz Case: An Incidentally Detected Mass of the Uterine Cervix. Mesonephric Adenocarcinoma of the Cervix. Arch. Pathol. Lab. Med..

[43] Stewart C.J., Taggart C.R., Brett F., Mutch A.F. (1993). Mesonephric Adenocarcinoma of the Uterine Cervix with Focal Endocrine Cell Differentiation. Int. J. Gynecol. Pathol..

[44] Lang G., Dallenbach-Hellweg G. (1990). The Histogenetic Origin of Cervical Mesonephric Hyperplasia and Mesonephric Adenocarcinoma of the Uterine Cervix Studied with Immunohistochemical Methods. Int. J. Gynecol. Pathol..

[45] Valente P.T., Susin M. (1987). Cervical Adenocarcinoma Arising in Florid Mesonephric Hyperplasia: Report of a Case with Immunocytochemical Studies. Gynecol. Oncol..

[46] Buntine D.W. (1979). Adenocarcinoma of the Uterine Cervix of Probable Wolffian Origin. Pathology.

[47] Rosen Y., Dolan T.E. (1975). Carcinoma of the Cervix with Cylindromatous Features Believed to Arise in Mesonerphric Duct. Cancer.

[48] Hart W.R., Norris H.J. (1972). Mesonephric Adenocarcinomas of the Cervix. Cancer.

[49] Tóth F., Csömör S., Mészáros J. (1964). Mesonephric Adenocarcinoma and Adenoma of the Cervix. Am. J. Obstet. Gynecol..

[50] Zaczek T. (1963). Mesonephric Carcinoma of the Cervix Uteri in an 11-Month-Old Girl Treated by Hysterectomy. Am. J. Obstet. Gynecol..

[51] McGee C.T., Cromer D.W., Greene R.R. (1962). Mesonephric Carcinoma of the Cervix—Differentiation from Endocervical Adenocarcinoma. Am. J. Obstet. Gynecol..

[52] Rose D. (1960). Cervical Adenocarcinoma of Mesonephric Origin. N. Engl. J. Med..

[53] Howitt B.E., Nucci M.R. (2018). Mesonephric Proliferations of the Female Genital Tract. Pathology.

[54] Burd E.M. (2003). Human Papillomavirus and Cervical Cancer. Clin. Microbiol. Rev..

[55] Tjalma W.a.A. (2019). HPV Negative Cervical Cancers and Primary HPV Screening. Facts Views Vis. ObGyn.

[56] da Silva E.M., Fix D.J., Sebastiao A.P.M., Selenica P., Ferrando L., Kim S.H., Stylianou A., Paula A.D.C., Pareja F., Smith E.S. (2021). Mesonephric and Mesonephric-like Carcinomas of the Female Genital Tract: Molecular Characterization Including Cases with Mixed Histology and Matched Metastases. Mod. Pathol. An. Off. J. United States Can. Acad. Pathol..

[57] Wu H., Zhang L., Cao W., Hu Y., Liu Y. (2014). Mesonephric Adenocarcinoma of the Uterine Corpus. Int. J. Clin. Exp. Pathol..

[58] Howitt B.E., Emori M.M., Drapkin R., Gaspar C., Barletta J.A., Nucci M.R., McCluggage W.G., Oliva E., Hirsch M.S. (2015). GATA3 Is a Sensitive and Specific Marker of Benign and Malignant Mesonephric Lesions in the Lower Female Genital Tract. Am. J. Surg. Pathol..

[59] Kenny S.L., McBride H.A., Jamison J., McCluggage W.G. (2012). Mesonephric Adenocarcinomas of the Uterine Cervix and Corpus: HPV-Negative Neoplasms That Are Commonly PAX8, CA125, and HMGA2 Positive and That May Be Immunoreactive with TTF1 and Hepatocyte Nuclear Factor 1-β. Am. J. Surg. Pathol..

[60] Pors J., Cheng A., Leo J.M., Kinloch M.A., Gilks B., Hoang L. (2018). A Comparison of GATA3, TTF1, CD10, and Calretinin in Identifying Mesonephric and Mesonephric-Like Carcinomas of the Gynecologic Tract. Am. J. Surg. Pathol..

[61] Park K.J. (2020). Cervical Adenocarcinoma: Integration of HPV Status, Pattern of Invasion, Morphology and Molecular Markers into Classification. Histopathology.

[62] Cibula D., Pötter R., Planchamp F., Avall-Lundqvist E., Fischerova D., Haie Meder C., Köhler C., Landoni F., Lax S., Lindegaard J.C. (2018). The European Society of Gynaecological Oncology/European Society for Radiotherapy and Oncology/European Society of Pathology Guidelines for the Management of Patients with Cervical Cancer. Radiother. Oncol..

[63] Sedlis A., Bundy B.N., Rotman M.Z., Lentz S.S., Muderspach L.I., Zaino R.J. (1999). A Randomized Trial of Pelvic Radiation Therapy versus No Further Therapy in Selected Patients with Stage IB Carcinoma of the Cervix after Radical Hysterectomy and Pelvic Lymphadenectomy: A Gynecologic Oncology Group Study. Gynecol. Oncol..

[64] Na K., Kim H.-S. (2019). Clinicopathologic and Molecular Characteristics of Mesonephric Adenocarcinoma Arising from the Uterine Body. Am. J. Surg. Pathol..

[65] Montagut C., Mármol M., Rey V., Ordi J., Pahissa J., Rovirosa A., Gascón P., Mellado B. (2003). Activity of Chemotherapy with Carboplatin Plus Paclitaxel in a Recurrent Mesonephric Adenocarcinoma of the Uterine Corpus. Gynecol. Oncol..

[66] Praiss A.M., White C., Iasonos A., Selenica P., Zivanovic O., Chi D.S., Abu-Rustum N.R., Weigelt B., Aghajanian C., Girshman J. (2024). Mesonephric and Mesonephric-like Adenocarcinomas of Gynecologic Origin: A Single-Center Experience with Molecular Characterization, Treatment, and Oncologic Outcomes. Gynecol. Oncol..

[67] Memorial Sloan Kettering Cancer Center (2024). Single Arm Phase II Study of Avutometinib (VS-6766) and Defactinib in Advanced or Recurrent Mesonephric Gynecologic Cancer. https://clinicaltrials.gov/study/NCT05787561.

[68] Gratias E.J., Dome J.S. (2008). Current and Emerging Chemotherapy Treatment Strategies for Wilms Tumor in North America. Paediatr. Drugs.

[69] Hoda S.A., Hoda R.S. (2020). Robbins and Cotran Pathologic Basis of Disease. Am. J. Clin. Pathol..

[70] Sadler T.W. (2022). Langman’s Medical Embryology.

[71] Gadd S., Huff V., Walz A.L., Ooms A.H.A.G., Armstrong A.E., Gerhard D.S., Smith M.A., Auvil J.M.G., Meerzaman D., Chen Q.-R. (2017). A Children’s Oncology Group and TARGET Initiative Exploring the Genetic Landscape of Wilms Tumor. Nat. Genet..

[72] Vaginal Agenesis and the Embryology of Vaginal Epithelium—Eizenberg—1994—Australian and New Zealand Journal of Obstetrics and Gynaecology—Wiley Online Library. https://obgyn.onlinelibrary.wiley.com/doi/abs/10.1111/j.1479-828X.1994.tb01112.x.

[73] Acién P. (1992). Embryological Observations on the Female Genital Tract. Hum. Reprod..

[74] Querleu D., Morrow C.P. (2008). Classification of Radical Hysterectomy. Lancet Oncol..

[75] Querleu D., Cibula D., Abu-Rustum N.R. (2017). 2017 Update on the Querleu-Morrow Classification of Radical Hysterectomy. Ann. Surg. Oncol..

[76] Querleu D., Cibula D., Abu-Rustum N.R., Fanfani F., Fagotti A., Anchora L.P., Ianieri M.M., Chiantera V., Bizzarri N., Scambia G. (2024). International Expert Consensus on the Surgical Anatomic Classification of Radical Hysterectomies. Am. J. Obstet. Gynecol..

[77] Muallem M.Z. (2021). A New Anatomic and Staging-Oriented Classification of Radical Hysterectomy. Cancers.

